# The meaning of sedentary behavior among older adults: a phenomenological hermeneutic study

**DOI:** 10.1186/s12889-023-16052-5

**Published:** 2023-06-13

**Authors:** Joakim Niklasson, Cecilia Fagerström, Patrick Bergman, Terese Lindberg, Sofia Backåberg

**Affiliations:** 1 Faculty of Health and Life Sciences, Linnaeus University, Kalmar, Sweden; 2 Department of Research, Region Kalmar County, Kalmar, Sweden; 3 Faculty of Health and Life Sciences, Department of Medicine and Optometry, Linnaeus University, eHealth Institute, Kalmar, Sweden; 4 Department of Health, Blekinge Institute of Technology, Karlskrona, Sweden; 5 Faculty of Health, and Life Sciences, Linnaeus University, Växjö, Sweden; 6 Faculty of Kinesiology, University of Calgary, Calgary, Canada

**Keywords:** Ageing, Frailty, Lived experience, Ordinary housing, Physical activity sedentary behavior

## Abstract

**Background:**

A sedentary lifestyle has implications for health and well-being. For healthy ageing, it is recommended to interrupt prolonged sitting; however, little is known about the meaning of sedentary behavior among older adults. The aim of this study was to understand the meaning of sedentary behavior among older adults with initial support from community care.

**Methods:**

A phenomenological hermeneutics approach was used, and individual interviews were conducted with sixteen older adults aged 70 to 97 years, by phone and face to face. The older adults lived in ordinary housing in southern Sweden and received initial support from community care.

**Results:**

The interviews yielded three key themes: Being sedentary is an unnatural part of life, having an ageing body means unwanted frailty, and having a sedentary lifestyle is based on conscious choices.

**Conclusion:**

Being sedentary means having a lack of physical activity and social interactions, resulting in wanting to be more physically active than sometimes possible. Clinical practitioners should bear in mind that becoming more sedentary is inevitable with an ageing body, but that older adults may have an innate desire to be as physically active as possible. A lifelong exposure to physical activity, the possibility of well-being found in sedentary activities and the impact of social networks should not be overlooked when creating clinical interventions to break unhealthy sedentary behavior among older adults. To increase the understanding of sedentary behavior among older adults, future research could focus on the impact of physical impairment on sedentary behavior and the relationship between sedentary behavior and physical activity throughout life.

**Supplementary Information:**

The online version contains supplementary material available at 10.1186/s12889-023-16052-5.

## Background

Through continuous development of health care, the possibility to prevent, treat, and cure diseases has increased. As a result, the number and proportion of adults aged over 60 years is increasing. Even with access to advanced medicine, the health impact of ageing appears as increased risk of chronic diseases, frailty, and developing psychological or physiological disorders [1]. The demographic shift, with more frail older adults and fewer people able to care for them, creates the need for strategies to promote healthy ageing [1]. To promote healthy ageing and prevent illness, it is recommended to be physically active for at least 150 min a week and to reduce prolonged sitting [2]. Sedentary behavior is defined as any activity, while awake, in a sitting or reclining posture, with energy expenditure ≤ 1.5 metabolic equivalents [3]. A systematic review by Harvey, Chastin [4] showed that older adults spend 60% (8.5–9.6 h) of their time awake sitting. Sedentary behavior among older adults is related to daily activities such as knitting, sewing, computer usage, playing cards, watching television, and going out to eat with friends [5, 6]. In promoting healthy ageing among older adults, identifying age-related deterioration at an early stage may help preserve physical function [1].

To be sedentary as an older adult is to experience pain, bodily aches, suffering, and lack of energy, often resulting in poor health [6–8]. According to a systematic review by Compernolle, De Cocker [9], older adults described physical inactivity as synonymous with sedentary behavior, knowledge regarding sedentary behavior was lacking, and there was a perception of when activities were expected to be sedentary. If a chosen activity was found to be convenient and/or enjoyable, this might justify sitting without feeling remorse [9]. These perceptions are important to understand when targeting the meaning of sedentary behavior, since they highlight motivational aspects of such behavior [9, 10]. In addition to embodied experiences, social relationships and physical environment have a large impact on sedentary behavior [11]. For example, experiencing expectations to sit more has been found to be a result of having family members or friends encourage older adults to sit more, based on lack of knowledge [8, 11]. According to Greenwood-Hickman, Renz [8], bad weather and reduced possibility to find a place to rest while walking have been shown to be reason enough to avoid physical activity.

Palmer, Gray [12] add new dimensions regarding justifications and excuses related to time spent sitting, especially as regards the importance of lived experiences in the public health approach to sedentary behavior among older adults. Among older adults, justifying sedentary behavior was described as a conscious moral act, and they took responsibility for time spent sitting. Regarding giving excuses for being sedentary, the findings of Palmer, Gray [12] describe this as an act of distancing from responsibility by implying that sedentariness is accidental, and thereby beyond the control of older adults. Future research needs to take stigmatization of sedentary behavior into account when striving for new understanding [12].

As previous studies have focused mainly on quantifying sedentary behavior using objective or self-reported measures [13], they have left a knowledge gap regarding the meaning of sedentary behavior [14–16]. If we reach a deeper understanding of the lived experience, the underlying psychological requirements for sustainable behavior change might be revealed [17]. Furthermore, it is important to reach an understanding of older adults’ lived experience of sedentary behavior since interventions are not currently designed in a manner that leads to sustainable behavior change and people tend to relapse into their old habits [18].

Today, there are few studies focusing on the lived experience of sedentary behavior, especially in older populations and, as McGowan, Powell [5] imply, there is a need for more in-depth knowledge to understand how to make sustainable behavior change among older adults. Thus, highlighting the importance of qualitative studies designed to increase awareness and more so to gain a deeper understanding of the meaning of sedentary behavior [5]. To our knowledge, the debate on how to interrupt or reduce sitting or how to increase physical activity in old age is ongoing, but has not covered how older adults understand sedentary behavior based on their lived experiences, which is the focus of the current study.

## Aim

To understand the meaning of sedentary behavior among older adults in an early stage of age-related health deterioration.

## Method

This study had a qualitative design based on individual interviews conducted with a phenomenological hermeneutical approach inspired by Ricœur [19], as described by Lindseth and Norberg [10], with the purpose to disclose truths about being sedentary as an older adult, based on the participants’ lived experiences. The method was used to address the possibility of interpreting more than one truth of the essential meaning of being in the life world, thus striving for a surplus of meanings [19].

### Participants

The participants were community-dwelling residents living in a municipality in southern Sweden, aged 71–90 years old. They were recruited from a larger, cross-sectional questionnaire study, *Sedentary behavior in older adults and supportive methods to promote healthy ageing*, conducted in 2018–2019. Out of the total population (1,617), 917 answered the questionnaire. The questionnaire was conducted in a region with a diversity of small towns and rural areas, where 24% of the citizens were 65 years or older and 11.5% received support from community care [20].

To be included in the current study, the participants had to receive initial support, defined as food distribution and/or security alarms from municipal caregivers. Residents with known cognitive impairment (e.g., diagnosed with dementia) were excluded from the study. All the 917 questionnaire respondents from baseline had the opportunity to participate in the current study by signing an interest form at the end of the questionnaire. Out of the 917 respondents, thirty older adults showed interest in participating in an interview follow-up. Of these thirty older adults, sixteen were randomly selected and contacted for participation in the current study. More in-depth information on the study was sent by post to the participants and informed consent for participation was obtained. The participants (identified as A–P) were nine women (average age 78 years, range 71–90) and seven men (average age 79 years, range 71–89; Table 1).

**Table 1:** Participants

**Id**	**Age ** **(years)**	**Social status and residence**	**Sex**	**Indoor activity**	**Outdoor activity**
A	81	Living alone in an apartment	Male	with the support of a walker	with the support of a walker
B	84	Living alone in an apartment	Female	with the support of a cane	with the support of a walker
C	72	Living alone in a house	Female	without walking aids	with the support of a walker
D	90	Living alone in a house	Female	without walking aids	with the support of a walker
E	74	Living alone in a townhouse	Female	with the support of a cane	with the support of a cane
F	73	Cohabiting in a house	Male	with the support of a walker	with an electric wheelchair
G	72	Cohabiting in a townhouse	Female	without walking aids	without walking aids
H	79	Living alone in an apartment	Male	without walking aids	without walking aids
I	84	Cohabiting in an apartment	Male	with the support of a cane	with the support of a cane
J	71	Living alone in an apartment	Male	with the support of a cane	with the support of a walker
K	89	Living alone in a house	Male	without walking aids	with the support of a walker
L	83	Living alone in a house	Female	with the support of another person	With the support of a walker
M	72	Living alone in a house	Female	without walking aids	without walking aids
N	80	Living alone in a house	Female	without walking aids	without walking aids
O	71	Living alone in an apartment	Female	without walking aids	with the support of a walker
P	77	Living alone in a house	Male	with the support of a walker	with the support of a walker

### Interviews

The interviewer (JN) contacted the participants and data were collected from March 2020 to April 2020 through individual in-depth interviews. The interviewer (JN) has clinical expertise within geriatrics and occupational therapy. The interviews were conducted with open-ended questions that allowed the participants to express their thoughts and understandings in their own words, which created a reflective habitat for the dialogue [21]. Each interview was opened with the question “What does it mean to be sedentary in old age?” followed by probing questions focused on the participant’s lived experiences. The interviewer continuously confirmed the interviewees’ stories by summarizing and retelling them throughout the respective interviews, to ensure that the interviewees’ stories were collected and not interpreted. Significant words were repeated, and pauses were used during the interviews to confirm understanding and obtain a full narrative. The number of participants was determined based on when no new information emerged in the interviews, as recommended by Lindseth and Norberg [10]. Due to restrictions related to the COVID-19 pandemic, eleven of sixteen interviews were held over the phone. The length of the interviews varied, with the shortest being 18 min and the longest 75 min (average 48 min) but the interviewer always strived to give the possibility of reaching the lived experience of sedentary behavior, searching for the ontological meaning. The interviews were recorded and transcribed verbatim.

### Data analysis

All authors read the transcribed interview texts. The first author (JN) made a preliminary analysis which was shared within the group, after which the interpretations were discussed. Dialogues regarding interpretation were conducted over time until consensus was reached. The interpretation process was inspired by Ricœur [19], moving between understanding the parts of the text, rebuilding the parts into whole passages of text, and ultimately reaching comprehension through explanation. The analysis process consisted of three overlapping steps, described by Lindseth and Norberg [10] as naïve reading, structural analysis, and comprehensive analysis.

In the naïve reading of the interviews, we entered a state of openness, shifting from a natural to a phenomenological attitude. To make this shift and be open to the understandable meaning implicit to the experience of sedentary behavior among older adults,, we had to refrain from listening to our own thoughts and judgments regarding the phenomenon [10]. The interview text was read as a whole, resulting in a naïve understanding of it and the meaning of the phenomenon. Continuous dialogue within the research group ensured that the group members’ pre-understandings did not guide the analysis process.

NVivo [22] was used to process the interview text for the structural analysis by condensing meaning units in the text, creating subthemes and themes. The whole research group validated the themes and subthemes, settling on three themes and eight subthemes. The themes were reflected on in relation to the naïve understanding and connections between the naïve understanding and the themes were uncovered.

The comprehensive analysis was used as a critical synthesis of the results. The synthesis was interpreted in relation to philosophical hermeneutic writings, to guide the interpretation of discourse by balancing reflection with imagination and also to navigate back and forth between surplus of meaning and fusion of horizons [19, 23–25] and recent research within the field of interest [10]. The process continued until consensus of the comprehensive understanding was reached, which opened for a immersed analysis of the text, in accordance with the chosen methodology [10].

## Results

### Naïve understanding

Sedentary behavior among older adults meant slowly but surely accepting the inevitable changes that ageing has on lifestyle, physical capabilities, and choices in daily life (Table 2). The awareness of the ageing body’s needs appeared to originate from a greater need for recovery and rest, as well as a feeling of being lazy, which it was made clear was based on a lack of discipline. Sedentary behavior was described as based on prevailing expectations of capabilities and active choices guided by interests and could occur both in solitude and together with other people. Having routines in everyday life was important and depending on the type of routines a person created, a more sedentary or active everyday life would be the result.

**Table 2:** Overview of themes and subthemes in older adults’ experiences of sedentary behavior in everyday life

Themes	Sub-themes		
Being sedentary is an unnatural part of life.	Having an inner drive for physical activity Norms emerging in encounters with others Striving for well-being through physical activity		
Having an ageing body means unwanted frailty	Having bodily restrictions creates discomfort		
	Losing authority over the body		
Having a sedentary lifestyle is based on conscious choices	Justifying everyday routines Hiding behind reasons to sit still		

### Being sedentary is an unnatural part of life

A sedentary lifestyle was experienced as unnatural and linked to expected levels of physical activity. Being physically active was considered as natural as breathing, but was affected by the natural process of ageing, leading to the experience of sedentary behavior. Being driven by a perceived need to be physically active in everyday life created a distant relationship to sedentary behavior.

#### Having an inner drive for physical activity

Inheriting an inner drive for physical activity created a distance to accepting becoming sedentary, since sedentary behavior was attributed to other people who were generally sedentary. Having this drive made sedentary behavior feel foreign. The drive to be active was based on an inner desire to treat the body in a healthy manner, sometimes with embedded guilt due to family members’ expectations to be more physically active to increase/maintain physical function. An inherent drive to be active could also have a basis in having grown up in a family where everyone was highly physically active. The inner drive to be active seemed to be strongly colored by others’ expectations: a feeling that one had to be active, since nothing else was acceptable. This strong belief regarding what the body needed became a solid ground for argumentation when the need for rest was mentioned.

*“Yes, you must keep your body moving, the body is made to be active. And needs it … actually, that is just the way it is. Everyone around me says that we are not made to sit on a chair, we need to get up and move around.” (B)*.

#### Norms emerging in encounters with others

Being sedentary meant that demands originating from relationships had an impact on the attitude towards daily physical activity. Observing peers being physically active or interacting socially could lead to physical activity but was not always a positive experience – potentially resulting in feeling shame and a need for improvement. Meetings with others also created a need to compare physical activity habits; such interactions led to thoughts on what was normal and not. Being physically active was not always experienced as a common behavior. If it was defined by family and friends as divergent from the expected way to behave, the behavior was seen as abnormal.

*“No, no, no, no, they are normal, everyone I hang out with, and my family has been completely normal. I’m the one who … and my husband, who has been very different, because we have always been active.” (A)*.

Social interaction described as the collisions of norms could lead to co-creation of meaning through being involved in someone else’s physical activity. In these interactions, being physically active was highly valued, while sedentary behavior was seen as the creator of deteriorating health.

#### Striving for well-being through physical activity

Engaging in preferred physical activities created well-being. Preferred physical activity was described as any activity providing well-being, and this affected priorities in daily life. Such prioritization took the form of breaking free from activities that involved sitting and engaging in outdoor activities that brought joy. However, not only a view on what was fun to do affected the choice of daily physical activities, but also a striving for feeling at peace and experiencing a state of absolute relaxation. This created a feeling of comfort and security.

*“No, I’m not in pain … I’m not in pain when I’m out walking … I walk a lot in the woods and stuff, and, like, it becomes a completely … a peace of mind, like, you think in a completely different way when your kind of do not have a lot of … impressions everywhere.” (J)*.

### Having an ageing body means unwanted frailty

Being sedentary meant having a frail, ageing body. The feeling of discomfort during physical activity and the impossibility of changing the course of nature created a permanent crack in what had been a familiar everyday life. This crack was a reminder of how to relate to an ageing body by adapting and compromising in everyday activities, resulting in sometimes having to face an unfamiliar need for rest.

#### Having bodily restrictions creates discomfort

Discomfort was described as pain limiting the ageing body’s natural movement patterns. Feeling pain when moving was not the only source of discomfort in the ageing body – living with a disease tended to prevent physical activity. Living with an ageing body, disease, and pain was a strong source of fear of hurting oneself while being active. Being active was no longer something fun and past priorities were often set aside and replaced by a feeling of no longer being able to rely on one’s body.

*“No, not … Now I’ve had … to stop // riding my bike // because I am scared, it’s because of my legs, right, when you’re getting on and off and when I was getting off, my leg gave way once, so I fell, and after that it was over.” (D)*.

#### Losing authority over the body

The need for recovery after getting tired and before continuing physical activity left an uneasy feeling of failing to understand the own body’s needs. Not understanding these needs was connected to remembering the former capabilities of a younger body, which caused a feeling of not being allowed to take it easy sometimes. This dissonance between a need for rest and productivity led to an ongoing struggle and required daily compromises. If a compromise was not balanced and resulted in exhaustion, there was a need for more rest than in the past. The ageing of the body changed the limits for physical activity, leading to the need to become familiar with these new conditions.

*“… you are older, you are more tired and maybe listless and … don’t have to get up and go.” (O)*.

### Having a sedentary lifestyle is based on conscious choices

Being sedentary was considered to encompass in activities while sitting or standing, but also some sedentary activities involved physical movement. Whether or not to be sedentary was a conscious choice faced multiple times a day and being able to make such a choice, created freedom. Being free to choose to sit meant having the right to do so, which gave the older adult a feeling of accepting their body. Even with this freedom, the ageing body had inevitable effects on everyday life, altering how or when physical activity was possible. The ability to take responsibility for being physically active was affected, replaced with an inability to choose a preferred physical activity, and a justification of sedentary behavior.

#### Justifying everyday routines

Everyday routines created a roadmap for navigating each day, which affected physical activity levels. Being active or not during the day was tied to everyday routines, which were often based on accustomed ways of thinking about sedentary behavior and the need for physical activities and sometimes justified prolonged sedentary behavior. Being able to choose to be physically active was grounds for arguments that sitting did not always equate to sedentary behavior, giving greater depth to the meaning of sedentary behavior.

*“No. And usually, when I’m sitting, I’ll be doing something, I’ll have lots of things on the table in front of me, newspapers or something, books that I’m going to read or cookbooks and then I’ll be looking for recipes in them and then you’ll be physically active too with … Also, with the arms and body.” (E)*.

#### Hiding behind reasons to sit still

Feeling unable to change physical activity patterns, or not feeling a need to do so, was shaped by excuses, distancing the older adult from taking responsibility for time spent sitting over the course of a long life. The distancing was based on feeling unable to change what was to come and was excused based on obstacles created by forces beyond one’s control. Reminiscing about a physically active past was sometimes seen as creating a right to sit still – if a person did not feel like being as active or felt unable to move as much as they used to.

*“Lazy. Yes, that … that is what I have become. I used to be … I never sat around, I was always on the go, but I’m not. I’m eighty-nine years old.” (P)*.

### Comprehensive understanding

Sedentary behavior in old age meant a lack of activity. The lifelong experience of movement, activity, and being physically active created a form of rearview mirror, showing a reflection of how the older adult had been able to be active and seemed to set a standard for “normal” sedentary behavior.

The view on sedentary behavior underwent a shift related to the ageing body’s limitations resulting from pain and disease, which had an impact on the capability to be as physically active as one saw fit. However, even though an ambition to be physically active was dominant, sedentary activities could triumph if well-being was found in them. The memories of a younger and healthier body led to a state of embodied awareness of no longer being fully in control of the body, resulting in an acceptance of no longer being able to do preferred physical activities, something that in the past had been taken for granted. This affected the unconscious balance between the actions that older adults believed were required to maintain health, their responsibilities, and their wishes [26]. Wishes and responsibilities involved not becoming more sedentary, which was a prerequisite for living a natural life and a constant feature in everyday life. There was a daily struggle between gaining peace through physical activity and the ageing body’s frailty. The ageing body’s frailty had a veto on what was possible and not, having a significant impact on how much time was spent sedentary. The capability of balancing the desire to be active with the need to sit still seemed to rely on the amount of exposure to physical activity throughout life.

Each person’s unique relationship with sedentary behavior had been shaped through social interactions, with each interaction contributing embodied knowledge. The cumulated experiences of sedentary behavior as an unnatural lifestyle, living with a frail ageing body and making sedentary choices in daily living brought new meanings to being sedentary. As described by Gadamer [25], this can be seen as widening one’s *horizon of understanding* through embodied knowledge, which occurs when pre-understanding collides with a new experience. The new experience countered pre-understood relationships and the embodied knowledge shaped a new understanding of how to relate to physical activity and sedentary behavior. Being sedentary meant having the right to decide to be active or not – thus, it was not a question of understanding the impact of physical activity, as this is well-known. Rather, it was about the possibility to be physically active or not in relation to prioritized activities in daily living. The striving to be as physically active as desired was constant, yet affected by unwanted frailty and daily habits, justifying sitting still.

## Discussion

This phenomenological hermeneutic study reveals the meaning of “sedentary behavior” among older adults and opens a discussion on its relationship to “physical activity.” Sedentary behavior is not yet fully conceptualized and relating to an unfamiliar way to behave is hard. When a situation was experienced as life-threatening or life-changing, such as being forced by a fragile body to become more sedentary, the relationship to the known body was altered. However, sedentary behavior in old age was about more than being physically inactive in terms of energy consumption measured in metabolic equivalents. It is interesting to consider that physical activities of low intensity such as walking were described as sedentary rather than as physical activity, which could be explained by the lived body’s experience of physical capacities [27]. The experiences gained within the life world were often related to the past and thus might not be as relevant for an older adult’s capabilities and their ageing body. The challenge related to describing “sedentary behavior” has been shown in past research striving to achieve consensus – but not entirely succeeding [3]. In our findings, the older adults described sedentary behavior as daily activities conducted while lying, sitting, or walking. This aligns with how older adults described sedentary behavior in the findings of McEwan, Tam-Seto [6].

In our findings being sedentary was an unnatural part of life measured against the expected daily physical performance. The nature of sedentary behavior was therefore distant but grew more familiar, because of the ageing body’s inability to replicate past performances. This created thoughts of what was possible and seemed to result in a feeling of having reduced possibilities to be active and losing the ability to independently perform everyday activities. One way to cope with the loss of autonomy in daily life was to rely on family and friends to get support. Needing to rely on others was a part of being sedentary and the relationship to caretaking has a vital role in how to cope with reduced physical capacity and a feeling of doing something wrong while sitting. The impact of having family members adopt a caretaking role seemed to create physical activity limitations and expectations of sedentary behavior, findings seen in previous research as well [11]. Our findings showed that when family and friends defined sedentary behavior as abnormal, and there was a conflict with how the older adult defined it, there was a risk of external pressures being applied. Though such pressures may break sedentary behavior, there is little or no guarantee for this leading to sustainable behavior change [17]. However, if the older adult became a caretaker, our findings showed that sedentary routines in daily living were willingly put aside for the important task at hand.

In our findings, the desire to be more physically active and that to reduce sedentary behavior were regarded in the same way, portrayed as interwoven and hard to separate from one another. These findings align with those of McGowan, Powell [5], who noted a resemblance between sedentary behavior and a lack of physical activity. Removing such an important part of life and not being able to achieve the expected physical activity levels due to an ageing body created a feeling of frailty.

For the older adults in this study sedentary behavior was connected to physical inactivity, viewed as a lack of physical activity or a desire to be more physically active than the frail ageing body allowed. Despite frailty, the ageing body was described as having a strong ability to adapt to changes within familiar physical activity patterns. Through the ageing body, a relationship to sedentary behavior could be created, with a range of factors affecting views on moving and sitting still. The ageing body increased acceptance of sedentary behavior, creating a shift to a more forgiving attitude as regards physical (in)activity and sedentary behavior. In a thematic synthesis by Rawlings, Williams [28], older adults described life slowing down with age, resulting in increased sedentary physical activities. The authors explained this as the result of both internal and external pressures. In our findings, internal pressures were experienced by older adults as a changing acceptance towards sedentary behavior, adapting to the ageing body. External pressures were felt when older adults did what was considered normal in their surroundings. Sedentary behavior was experienced as an ongoing, socially accepted bodily transformation. Distinguishing between external and internal factors may not always be easy and contextual circumstances should be specified in each individual situation to create a clearer understanding of a behavior [17].

The feeling of no longer being in charge and being reminded of the social assumption that older adults should adopt a more sedentary lifestyle has an impact on how older adults think they should behave [6, 11]. Following a lifetime of preferences regarding when or how to be physically active, the relationship to sedentary behavior is altered. In this study the experience of a past active life grants the capability of accepting life as it is. Not being able to move one’s body in the way one used to place the older adult’s body in a transformation stage, where the loss of physical capacity can be experienced and accepted as “becoming older.” This phase of accepting the ageing body alters the view of sedentary behavior as unnatural, resulting in a path forward in daily living.

Furthermore, negative effects of sedentary behavior were reflected on and acknowledged but ignored if other favored benefits were related to the chosen activity. In our findings playing cards, co-solving crossword puzzles, or just sitting and engaging in small talk with a friend or family member were vital breaks in the repetitive cycle of daily living. All those activities involved sitting – but if there was a chance of social interaction, the choice was easy. Meeting people was crucial to well-being, and if there was an opportunity to meet new people and gain social or cognitive benefits, this outweighed the risks and justified sedentary behavior. Similar findings have been noted in research by McEwan, Tam-Seto [6], but the same social priorities have also been shown to inspire more physical activity.

### Methodological considerations

The conditions for participating in the study were carefully chosen to ensure a representative sample with a variety in social status, age, sex, and physical capacity, which strengthened the dependability [29]. Most of the interviewees were able to move around independently, which was considered both a strength and a limitation. Although this created a risk of including a group consisting of highly active older adults, it was regarded as important that participants had lived experiences of physical activity, to achieve depth in the interviews. Thus, with all participants receiving initial support from community care, the lived experience of becoming more sedentary due to age-related deterioration would also be embedded in their life worlds [30].

When conducting interviews, there is always a risk of misunderstanding the narrative, since the interviewee and interviewer relate to their own unique preunderstandings. Therefore, there was discussion within the research group before analysis about bracketing of judgmental views or known facts about sedentary behavior. To prepare for the interviews the interviewer took research class in qualitative methods, focusing on interview technique and phenomenological hermeneutics methods. Acknowledging of preunderstandings was done to stay as open as possible to the understandable meaning found within the narratives of the lived experiences. All interviews were conducted by the same person, who had clinical expertise within the field. The study was conducted in part during the COVID-19 pandemic, meaning that most interviews were held over the phone. Not meeting in person could be expected to affect interviews in a negative way, but the interviewees spoke freely and the expected obstacle to getting a rich text was never experienced.

The first step of validation was to ensure the link between the naïve understanding, seen as an immediate life world perception, and the structure analysis [10]. The structure analysis was the result of a repeated validation process within the research team consisting of discussing alternative interpretations and descriptions of sedentary behavior. We also placed emphasis on discussing the differences and similarities between our findings and those from other settings, to achieve recognizability. This is a part of the validation methodology described as “shared ideas” and “universality” by Lindseth and Norberg [10], originating from Paul Ricœur’s terminology “surplus of meanings” [19]. Regular debriefing sessions were conducted within the research group to ensure a focus on the phenomenon and judgmental bracketing, and to understand multiple ways of interpreting the text, which Lindseth and Norberg [10] describe as an important part of validation.

The meaning of sedentary behavior gained unique inputs from each interview – a surplus of meanings – resulting in more than one way to understand sedentary behavior thanks to finding an intersubjectively comprehensible meaning of lived experience [10]. The connection between structure analysis and naïve understanding validates the analysis process and its reliability [10], a connection confirmed by all research group members in the current study.

A naïve understanding can be found within the comprehensive analysis of the current study, as well as a more profound understanding which provides new perspectives on the meaning of sedentary behavior, adding further insights. Going from interviews to a comprehensive understanding was a process serving to reveal what was disclosed in the text [24].

## Conclusions

Sedentary behavior among older adults is more than simply sitting still and not doing anything – and does not have to be related to a certain posture. Sedentary behavior means having a lack of physical activity, with the outcome of wanting to be more physically active than sometimes possible. The innate desire to be as physically active as possible was clear among those with an ageing body that required rest. The never-ending striving to be physically active appeared to be influenced by social interactions and choices in daily living. Thus, being sedentary was altered by preferences and could also mean having a lack of social interactions that encourage healthy activity behavior. Having been raised in a family where physical activity was a natural part of daily life made striving to be more physically active and maintain physical function natural to some older adults. Losing a functional body leads to a sense of how physical activity has been taken for granted throughout life. This changes the relationship to sedentary behavior, creating a need for acceptance of the ageing body’s frailty, and requires the older adult to adapt to a more unnatural sedentary lifestyle.

Being forced by an older body to become sedentary was not easy, a finding that healthcare workers should keep in mind when addressing recommendations set for physical activity. Clinical practitioners creating interventions to break unhealthy sedentary behavior would also benefit from not overlooking older adults’ lifelong exposure to physical activity, the benefits that sedentary behavior could bring to well-being, and the impact of social networks. The amount of physical activity throughout life was connected to sedentary behavior as an older adult and future research should focus on understanding this relationship in more detail. Future research would also be encouraged to include older adults at all physical impairment levels.

## Electronic supplementary material

Below is the link to the electronic supplementary material.

Supplementary Material 1

Supplementary Material 2

## Data Availability

All data generated or analyzed during this study are included in this published article [and its supplementary information files].
