# Supplementary material for: The meaning of sedentary behavior among older adults: a phenomenological hermeneutic study
Source: BMC Public Health. 2023 Jun 13;23:1134. doi: 10.1186/s12889-023-16052-5 (PMC10262142; doi:10.1186/s12889-023-16052-5)
Supplement: Supplementary file 1 — Supplementary Material 1 [file 12889_2023_16052_MOESM1_ESM.docx]

# Interview guide

1. What does it mean to be sedentary in old age?
2. What are your thoughts regarding being sedentary later in life?
3. What does the word sedentary make you think of?
4. What could help you reduce time spent sedentary?

Examples of narrative probing questions:

- ...and how did that make you feel?
- …what happened after that?
- …who is this person to you?
- …can you please elaborate?

Arching research questions to have in mind during the interviews:

How does the older adult describe the concept of sedentary behavior?

What experiences are there about the sedentary nature of other older adults?

What challenges do the older adult describe regarding breaking a sedentary lifestyle?

What is the older adult experience of existing support for a changed movement behavior?

What future needs regarding support to break/change a sedentary behavior do the older adult experience?
