# Supplementary material for: The meaning of sedentary behavior among older adults: a phenomenological hermeneutic study
Source: BMC Public Health. 2023 Jun 13;23:1134. doi: 10.1186/s12889-023-16052-5 (PMC10262142; doi:10.1186/s12889-023-16052-5)
Supplement: Supplementary file 2 — Supplementary Material 2 [file 12889_2023_16052_MOESM2_ESM.docx]

| **Topic guide** | | | |
| --- | --- | --- | --- |
| **Themes** | **Sub-themes** | | |
| Being sedentary is an unnatural part of life | Having an inner drive for physical activity  Norms emerging in encounters with others  Striving for well-being through physical activity | | |
|  |  | | |
| Having an ageing body means unwanted frailty | Having bodily restrictions creates discomfort | | |
|  | Losing authority over the body | | |
|  |  |  |  |
| Having a sedentary lifestyle is based on conscious choices | Justifying everyday routines  Hiding behind reasons to sit still | | |
